# Progress on Self-Powered Wearable and Implantable Systems Driven by Nanogenerators

**DOI:** 10.3390/mi12060666

**Published:** 2021-06-07

**Authors:** Lanxin Yang, Zhihao Ma, Yun Tian, Bo Meng, Zhengchun Peng

**Affiliations:** Key Laboratory of Optoelectronic Devices and Systems of Ministry of Education and Guangdong Province, College of Physics and Optoelectronic Engineering, Shenzhen University, Shenzhen 518060, China; yanglanxin2020@email.szu.edu.cn (L.Y.); mzh_930930@163.com (Z.M.); 1910454039@email.szu.edu.cn (Y.T.); zcpeng@szu.edu.cn (Z.P.)

**Keywords:** self-powered systems, nanogenerator, wearable electronics, implantable devices

## Abstract

With the rapid development of the internet of things (IoT), sustainable self-powered wireless sensory systems and diverse wearable and implantable electronic devices have surged recently. Under such an opportunity, nanogenerators, which can convert continuous mechanical energy into usable electricity, have been regarded as one of the critical technologies for self-powered systems, based on the high sensitivity, flexibility, and biocompatibility of piezoelectric nanogenerators (PENGs) and triboelectric nanogenerators (TENGs). In this review, we have thoroughly analyzed the materials and structures of wearable and implantable PENGs and TENGs, aiming to make clear how to tailor a self-power system into specific applications. The advantages in TENG and PENG are taken to effectuate wearable and implantable human-oriented applications, such as self-charging power packages, physiological and kinematic monitoring, in vivo and in vitro healing, and electrical stimulation. This review comprehensively elucidates the recent advances and future outlook regarding the human body’s self-powered systems.

## 1. Introduction

Wearable electronics and implantable devices have drawn much attention in academic research and industry [1]. However, the traditional wearable and implantable systems were bulky, with high replacement frequency and a short life span [2]. In recent years, with the contemporary increased demands of multiple and ubiquitous wearable and implantable applications, human-oriented self-powered systems have become a hot issue [3,4].

To this end, various technologies have been developed for continuous electricity supply of the wearable and implantable systems. Electrical energy could be transmitted or captured from the ambient environment or the human body itself [3]. Among these approaches, wireless power transmission [5], photovoltaic cells [6], and thermoelectricity [7] all rely too much on external conditions. Due to limited conditions, the utilization rate is not high enough, making them difficult to effectively utilize on a large scale.

The biomechanical energy produced by human motions and the round-the-clock biological rhythms could be a promising power source to realize self-powered wearable and implantable systems. Diverse mechanisms of energy harvesting have been developed to capture and convert this tiny, ubiquitous, neglected, and wasted biomechanical energy into electricity [3].

In 2006, Wang’s group developed a piezoelectric nanogenerator (PENG) based on ZnO nanowires [8,9], which brought a breakthrough to the miniaturization of energy harvesting. In 2012, Wang’s group invented the triboelectric nanogenerator (TENG) [10], which is a milestone discovery in energy harvesting and self-powered systems. Nanogenerators based on piezoelectric and triboelectric effects have the advantages of low cost [11], high efficiency [12], flexibility [13], light weight [14], and strong sustainability [15]. They are widely used as a power supplier in portable electronics, the internet of things, and human–machine interfaces, and as active sensors for engineering and environmental monitoring [16].

Meanwhile, nanogenerators were explored for biological use and quickly played an important role in the development of [17,18,19,20,21,22,23,24,25,26,27,28,29,30,31,32,33,34,35,36,37,38,39,40,41,42,43,44,45,46,47,48,49,50,51,52,53,54,55,56,57,58,59,60,61,62,63,64,65,66,67,68,69,70,71,72,73,74,75,76,77,78,79,80,81,82] and implantable [83,84,85,86,87,88,89,90,91,92,93,94,95,96,97,98,99,100,101,102,103,104,105,106,107,108,109,110,111,112,113,114,115,116,117,118,119,120] systems. In 2012, Minbaek Lee et al. demonstrated a hybrid nanogenerator composed of ZnO and PVDF, which stimulated the research of wearable PENGs [71]. In 2013, Xiaosheng Zhang et al. proposed a sandwich-shaped TENG and implemented the first demonstration of the nanogenerator to directly drive a biomedical microsystem [95].

**Figure 1:**
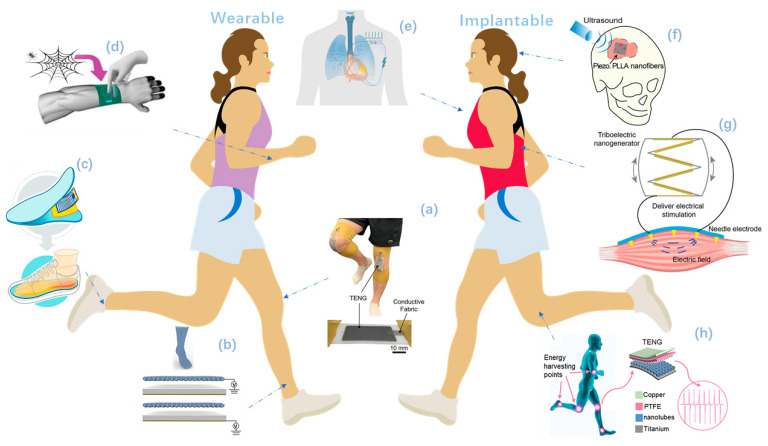
Overview of self-powered wearable and implantable systems driven by nanogenerators. (**a**–**d**) Self-powered wearable systems. (**a**) A stretchable liquid metal elastomer based TENG patch attached on the knee. Reproduced with permission from Ref. [60]. Copyright © 2020, Wiley-VCH. (**b**) A self-powered and self-functional cotton sock. Reproduced with permission from Ref. [32]. Copyright © 2019, American Chemical Society. (**c**) A commercial electric heating sheet powered by PENG. Reproduced with permission from Ref. [48] Copyright © 2020, American Chemical Society. (**d**) A bio-inspired spider-net-coding interface to detect and control multiple directions. Reproduced with permission from Ref. [55]. Copyright © 2019, Wiley-VCH. (**e**–**h**) Implantable self-powered systems: (**e**) A symbiotic cardiac pacemaker. Reproduced with permission from Ref. [101]. Copyright © 2019, Springer Nature. (**f**) A biodegradable, battery-less electrical stimulator made of piezoelectric nanofibers, serves as a bone scaffold. Reproduced with permission from Ref. [85]. Copyright © 2020, Elsevier. (**g**) Electrical muscle stimulation directly powered by TENG. Reproduced with permission from Ref. [98]. Copyright © 2019, Wiley-VCH. (**h**) A self-powered treatment to charge implant surface. Reproduced with permission from Ref. [99]. Copyright © 2020, Elsevier.

As briefly illustrated in Figure 1, nanogenerators have been employed to work in multiple parts of the human body. The TENGs and PENGs are integrated into wearable textiles and shoes [32,48] or mounted on human skin [55,60], serving as power supplies or active sensor for motion and vital signs monitoring [32,48,55,60]. In addition, the implanted nanogenerators, which are biocompatible and even biodegradable [85], play an influential role in biomedical applications to power implanted devices [99,101], to record biological signals, and to stimulate muscles and the nervous system in therapy use [85,98].

In this review, as illustrated in Table 1, we focus on a comprehensive overview of recent advances in self-powered wearable and implantable systems that are energized by nanogenerators. Through the development of self-powered systems, we summarize the optimization of materials and structures in wearable and implantable nanogenerators. Further, we expand on the applications of self-powered wearable and implantable systems.

## 2. Materials and Structural Design of Wearable and Implantable Nanogenerators

### 2.1. Materials of Wearable and Implantable Nanogenerators

#### 2.1.1. Materials of Wearable and Implantable TENGs

**Figure 2:**
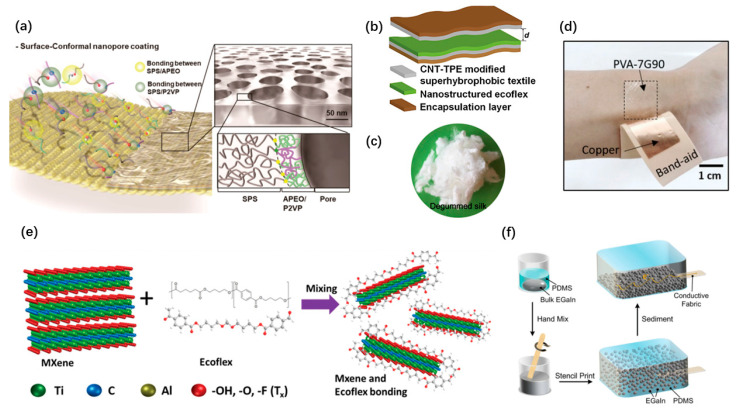
Materials of wearable and implantable TENGs. (**a**) Surface-conformal nanoporous films coated on textiles. Reproduced with permission from Ref. [59]. Copyright © 2020, American Chemical Society. (**b**) A textile TENG with super-hydrophobic coating. Reproduced with permission from Ref. [44]. Copyright © 2020, Wiley-VCH. (**c**) Natural silk fibers for a wearable TENG. Reproduced with permission from Ref. [61]. Copyright © 2020, Elsevier. (**d**) TENG built with biodegradable PVA gelatin. Reproduced with permission from Ref. [45]. Copyright © 2020, Wiley-VCH. (**e**) MXene/Ecoflex nanocomposite as a negative friction layer. Reproduced with permission from Ref. [56]. Copyright © 2020, Wiley-VCH. (**f**) Liquid metal elastomer composite for stretchable TENG. Reproduced with permission from Ref. [60]. Copyright © 2020, Wiley-VCH.

Owing to the extensively exiting of triboelectrification, TENGs have greater selectivity in materials. Surface modification on textiles is an efficient approach to obtain excellent and low-cost friction layers for wearable TENG. It has been widely studied [44,59]. Chanho Park et al. put forward a one-step route for developing rapid wet processable surface-conformal nanoporous films [59], as shown in Figure 2a, which are made up of a ternary polymer blend of sulfonic-acid-terminated poly(styrene), poly(2-vinylpyridine) and amine-terminated poly(ethylene oxide) in benzene. These mixed materials can result in well-defined nanopores. As shown in Figure 2b, Feng Wen et al. proposed a simple carbon nanotube (CNT)/thermoplastic elastomer coating method to achieve super hydrophobicity of the textile TENG [44]. Biocompatible and biodegradable materials, especially bio-absorbable natural materials such as wood, silk, wheat cotton, and cellulose, provide more opportunities for wearable and implantable applications. Meng Su et al. proposed a CNT-silk mixing layer as the conductive friction material to realize a wholly biodegradable TENG [53]. Qianqian Niu et al. adopted silk nanoribbons with adjustable sizes and stable aqueous conditions and developed an all-silk bio-TENG [61], as shown in Figure 2c. Moreover, biodegradable polymers such as polyvinyl alcohol (PVA) and polycaprolactone (PLC) were widely used. Ruoxing Wang et al. proposed a wearable TENG based on biodegradable PVA [45], as shown in Figure 2d. The fabricated PVA-gelatin composite film provides a choice for achieving skin-friendly TENG. Stretchability would be an essential requirement for specific wearable and implantable TENGs. Conductive 2D materials and liquid metals can help. Md Salauddin et al. presented a conductive fabric-based TENG, which is made up of MXene (Ti3C2Tx) nanosheets and Ecoflex composite [56], as shown in Figure 2e. Chengfeng Pan et al. presented an ultra-stretchable TENG based on the sedimented liquid metal elastomer composite [60], as shown in Figure 2f. It possesses excellent conductivity under ultrahigh stretchability.

#### 2.1.2. Materials of Wearable and Implantable PENGs

**Figure 3:**
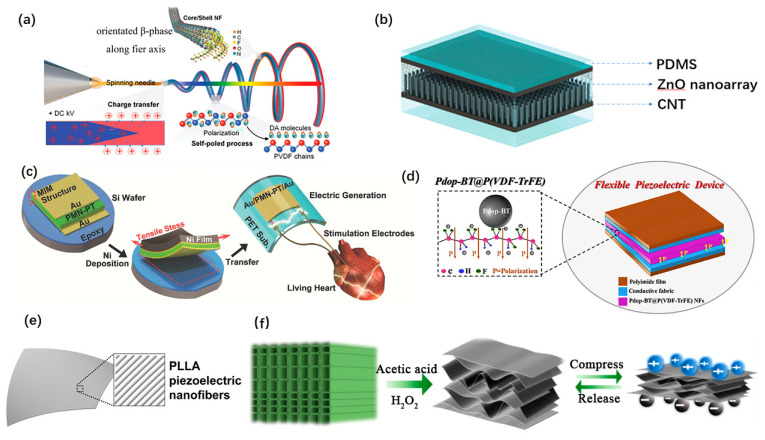
Materials of wearable and implantable PENGs. (**a**) A core/shell PVDF/dopamine nanofiber-based PENG. Reproduced with permission from Ref. [29]. Copyright © 2020, Wiley-VCH. (**b**) A zinc oxide nanoarrays based PENG. Reproduced with permission from Ref. [84]. Copyright © 2021, John Wiley and Sons. (**c**) A PENG based on PMN-PT. Reproduced with permission from Ref. [110]. Copyright © 2014, Wiley-VCH. (**d**) A BaTiO3@P(VDF-TrFE) nanocomposite-based PENG. Reproduced with permission from Ref. [76]. Copyright © 2020, Elsevier. (**e**) A biodegradable PENG based on PLLA nanofibers. Reproduced with permission from Ref. [118]. Copyright © 2020, National Academy of Sciences. (**f**) A PENG based on wood sponge. Reproduced with permission from Ref. [50]. Copyright © 2020, American Chemical Society.

Polyvinylidene fluoride (PVDF) and its copolymers are considered as the most promising candidates for wearable and implantable PENG due to their high flexibility, good biocompatibility and processability [41]. Tong Li et al. designed an all-fiber-based PENG using core/shell PVDF/dopamine (DA) nanofibers [29], as shown in Figure 3a. The use of a self-assembly process to form and arrange β-phase PVDF can further enhance the piezoelectric performance while maintaining excellent reliability. Kuntal Maity et al. reported a PENG pressure sensor based on highly aligned PVDF nanofibers arrays and achieved a high sensitivity of 0.8 V/KPa [63]. ZnO nanowire is widely used as well. Congran Jin et al. developed a PENG based on ZnO nanoarrays embedded in a PDMS membrane [84], as shown in Figure 3b. It generates 9.2 V open-circuit voltage and can be stretched to 250%. Typical lead-containing piezoelectric materials are toxic with poor mechanical properties. Well packaged lead-containing PENGs with flexible substrate could also achieve high performance in implantable applications. Geon-Tae Hwang et al. proposed a flexible PENG based on single-crystalline PMN-PT [110], as shown in Figure 3c. This PMN-PT has a piezoelectric charge constant of d_33_ up to 2500 pC/N. Composites of multiple organic and inorganic piezoelectric materials were studied to develop flexible PENGs with a high charge constant. Xiaoyang Guan et al. proposed a wearable, flexible PENG based on nanocomposite fibers [76], as shown in Figure 3d. This hierarchical micro-structured piezoelectric membrane is fabricated by electrospun P(VDF-TrFE) fibers with polydopamine modified BATiO_3_ nanoparticles anchored on the surface. Among the piezoelectric materials for wearable and implantable use, biocompatibility and biodegradability are the kernels to be considered. Eli J. Curry et al. Proposed a biodegradable and biocompatible poly(L-lactic acid) (PLLA) nanofiber with highly controllable and stable piezoelectric properties [118], as shown in Figure 3e. This PENG has shown good performance for implanted use. Jianguo Sun et al. developed a PENG based on the natural balsa wood [50], as shown in Figure 3f. The piezoelectric wood sponge is fabricated with a simple chemical delignification treatment on the natural wood. In addition, it can be decomposed with cellulose-degrading fungi.

### 2.2. Structures of Wearable and Implantable Nanogenerators

#### 2.2.1. Structures of Wearable Nanogenerators

**Figure 4:**
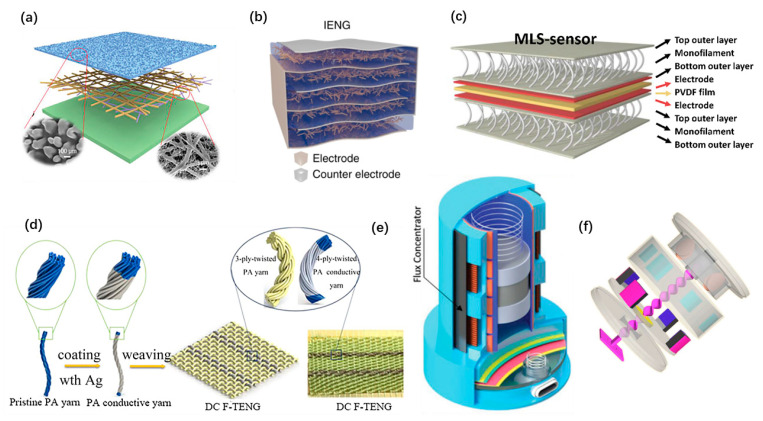
Structures of wearable nanogenerators. (**a**) An ultrathin skin-inspired TENG. Reproduced with permission from Ref. [74]. Copyright © 2020, Wiley-VCH. (**b**) A PENG with a three-dimensional intercalation electrode. Reproduced with permission from Ref. [77]. Copyright © 2020, Springer Nature. (**c**) A 3D textile structured PENG. Reproduced with permission from Ref. [49]. Copyright © 2020, Elsevier. (**d**) A textile TENG. Reproduced with permission from Ref. [81]. Copyright © 2020, American Chemical Society. (**e**) An inertial structured hybrid nanogenerator. Reproduced with permission from Ref. [64]. Copyright © 2020, Wiley-VCH. (**f**) A linear-to-rotary hybrid wearable nanogenerator. Reproduced with permission from Ref. [42]. Copyright © 2020, Elsevier.

The large number of materials enables theoretical models to be transformed into nanogenerators with various structures, which not only realizes the functions of the device, but also has the extra advantages of the materials. Benefiting from the outstanding flexibility, wearable nanogenerators can be easily designed as simple thin-film structures. These nanogenerators are generally attached on the skin. Yang Jiang et al. developed an ultrathin skin-like TENG [74], as shown in Figure 4a. It adopted a single-electrode structure with a stretchable and transparent electrode, and forms a comfortable and conformal device that can attach to the epidermis. Xiao Peng et al. proposed an all-nanofiber single-electrode TENG with a hierarchical porous structured friction film [58], which is stretchable, breathable, and biodegradable. Wearable nanogenerators existing as part of textiles, shoes, or other wearable accessories are prevalent as well. Multiple-layered plain structures and 3D textile structures were developed to improve the output performance of nanogenerators. Long Gu et al. proposed a PENG with a three-dimensional intercalation electrode [77], as shown in Figure 4b. It can charge a 1 μF capacitor from 0 V to 8 V in 21 cycles. Seongcheol Ahn et al. proposed a 3D textile structured PENG with pre-strained monofilament [49], as shown in Figure 4c. The 3D structure of the monofilament is employed as a pressure transmitter for piezoelectric amplification to improve the sensitivity. A direct current fabric TENG with a plain structure was proposed by Chaoyu Chen et al. [81], as shown in Figure 4d. It can produce high DC outputs to harvest the energy from the electrostatic breakdown phenomenon of clothes during human motions. Most of the energy generated by human motions is at low frequency and low acceleration [62]. Inertial structured spring-mass systems have been proved to be an efficient way to achieve high energy harvesting efficiency for wearable nanogenerators. They were usually designed as hybrid nanogenerators. Pukar Maharjan et al. proposed a wearable hybrid nanogenerator that shows high performance under low acceleration (≤1 g) and low frequency (≤6 HZ) human motions [64], as shown in Figure 4e. Cheng Yan et al. designed a linear-to-rotary hybrid nanogenerator to achieve high output performance by frequency enhancement [42], as shown in Figure 4f.

#### 2.2.2. Structures of Implantable Nanogenerators

**Figure 5:**
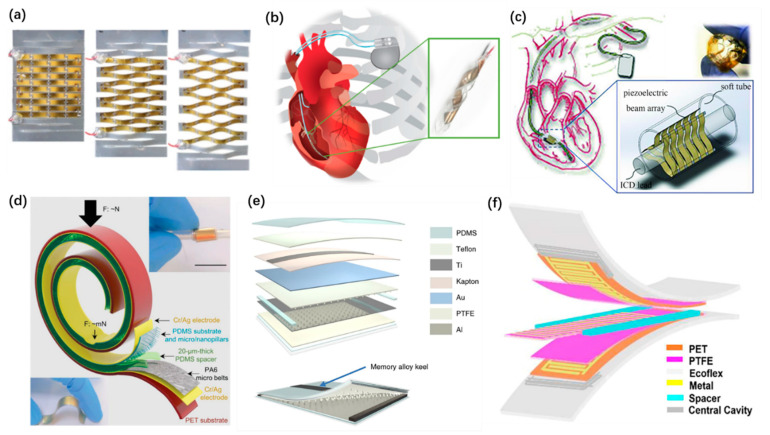
Structures of implantable nanogenerators. (**a**) A kirigami inspired PENG. Reproduced with permission from Ref. [83]. Copyright © 2019, Wiley-VCH. (**b**) A helix structured PENG. Reproduced with permission from Ref. [116]. Copyright © 2019, Elsevier. (**c**) A PENG with buckled beam array structure. Reproduced with permission from Ref. [114]. Copyright © 2018, Wiley-VCH. (**d**) A mechanically asymmetrical TENG. Reproduced with permission from Ref. [120]. Copyright © 2020, Wiley-VCH. (**e**) An implantable TENG with 3D sponge spacer. Reproduced with permission from Ref. [101]. Copyright © 2019, Springer Nature. (**f**) A stretchable micro-grating structured TENG. Reproduced with permission from Ref. [115]. Copyright © 2018, American Chemical Society.

For in vivo applications, the implantable nanogenerators usually employ thin-film structures and their transformation. Transformative thin-film structures are widely used in implantable PENGs. A circular piezoelectric belt is one of the simplest and effective ones [119]. Sophisticated designs of the transformation are implemented to obtain enhanced electrical outputs. Rujie Sun et al. proposed a kirigami stretchable structure of PENG [83], as shown in Figure 5a. It improves the tensile property and flexibility of PENG to implant on the organs and achieves much higher outputs than unstructured design. Lin Dong et al. developed implantable PENGs with a helix structure [116], as shown in Figure 5b and a buckled beam array design [114], as shown in Figure 5c. These PENGs deform through the movement of the pacemaker lead and generate stable electricity. Most of the implantable TENG adapt a contact-separation structure. Owing to the limited separation space in the body, the structures should be well designed to maintain the practical work of TENGs. Bolang Cheng et al. proposed a mechanically asymmetrical TENG [120], as shown in Figure 5d. A 20 µm thick PDMS spacer is used, and the TENG belt can be twisted and rolled up to different shapes. It can monitor the microscopically weak intestinal peristalsis. However, due to the low stiffness of the friction layers, the separation of implantable TENGs will be reduced, which may lead to decreasing output performance. To overcome this, an implantable TENG with a 3D sponge spacer was developed [101], as shown in Figure 5e. A memory alloy ribbon serving as the keel of the friction layer is employed, to obtain a higher long-term stability. Zhao Chaochao et al. fixed two magnets on the back of the friction layers to produce repulsion separation when contact occurs. Thus, the life cycle of the TENG is extended [94]. Well-designed sliding mode TENGs can also work well in vivo. Jun Li et al. reported a stretchable micro-grating structured TENG [115], as shown in Figure 5f. It was implanted inside a rat’s abdominal cavity to harvest energy from ventral diaphragm movement.

## 3. Self-Powered Wearable Systems

### 3.1. Self-Powered Wearable Systems Based on TENG

**Figure 6:**
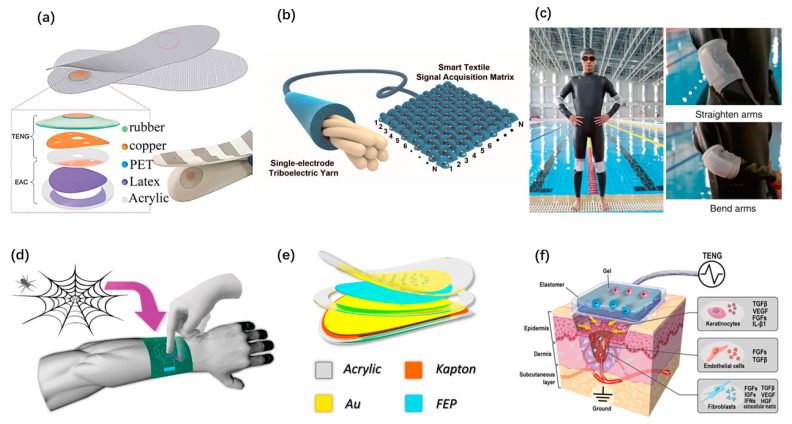
Self-powered wearable systems based on TENGs. (**a**) A TENG-based smart insole. Reproduced with permission from Ref. [69]. Copyright © 2020, Wiley-VCH. (**b**) An ultralight single-electrode triboelectric yarn with helical hybridized nano-micro core-shell fiber bundles. Reproduced with permission from Ref. [65]. Copyright © 2020, American Chemical Society. (**c**) A bionic stretchable TENG for underwater rescue. Reproduced with permission from Ref. [30]. Copyright © 2020, Springer Nature. (**d**) A bio-inspired spider-net-coding interface for multiple direction detecting and control. Reproduced with permission from Ref. [55]. Copyright © 2019, Wiley-VCH. (**e**) A self-powered auditory sensor with ultrahigh sensitivity. Reproduced with permission from Ref. [20]. Copyright © 2020, Elsevier. (**f**) A wearable ionic TENG patch for wound healing. Reproduced with permission from Ref. [75]. Copyright © 2020, Elsevier.

Wearable electronics have brought conveniences to our daily life. In the face of the increasing demand for self-powered wearable systems, TENGs have played an important role in active sensing and mechanical energy harvesting. Clothing is a necessity in our daily life. Putting the concept of wearable TENG on clothes is bound to be a hot issue pursued by researchers. Textile and fabric-based TENGs have been developed rapidly. Wenjing Fan et al. presented a textile TENG sensor array with high-pressure sensitivity [78]. This device is employed as a noninvasive method to evaluate the signal generated by cardiovascular disease and sleep apnea syndrome. Zhiming Lin et al. reported a smart insole with TENG embedded as an active sensor for real-time gait monitoring [69], as shown in Figure 6a. It has high durability and excellent mechanical robustness to monitor the abnormality of gait for rehabilitation assessment. Liyun Ma et al. proposed an ultralight single-electrode textile-based TENG with helical hybridized nano-micro core-shell fiber bundles [65], as shown in Figure 6b. It enables harvesting biomechanical energy and monitoring tiny signals from human motions. TENGs come in a wide range of forms besides fabrics. Yu Song et al. developed a self-powered wearable wireless sweat sensing system based on a TENG [54]. As shown in Figure 6c, Yang Zou et al. designed a bionic stretch TENG by imitating the electric eel’s power generation principle [30]. It has a broad application prospect in underwater motion detection and submarine rescue. With the advent of the intelligent era, objects are connected through the internet, and wearable applications for human–machine interfaces and intelligent systems are also arising. A smart glove with a haptic feedback was designed based on TENG to serve as a simple human–computer interaction method [31]. Qiongfeng Shi et al. reported a bio-inspired spider-net-coding interface with great flexibility and scalability [55]. By employing a single-electrode TENG, detection and control of multiple directions are demonstrated, as shown in Figure 6d. Hengyu Guo et al. proposed a self-powered acoustic sensor [20], as shown in Figure 6e. It created a new acoustic system by using TENG. The acoustic sensor has ultrahigh sensitivity, which could reach 110 mV/dB. Wearable TENGs are adopted for biomedical applications as well. Zhirong Liu et al. developed a TENG as a stable voltage pulse source to trigger plasma membrane potential and membrane permeability for intracellular drug delivery [67]. The delivery efficiency of this system is 90%, and the cell survival rate is more than 94%. Yonghong Li et al. devised a wearable ionic TENG, which has a stretchable gel composition [75]. The electricity generated by this TENG from biomechanical energy is used in damaged tissues, and it accelerates the wound healing, as shown in Figure 6f.

### 3.2. Self-Powered Wearable Systems Based on PENG

**Figure 7:**
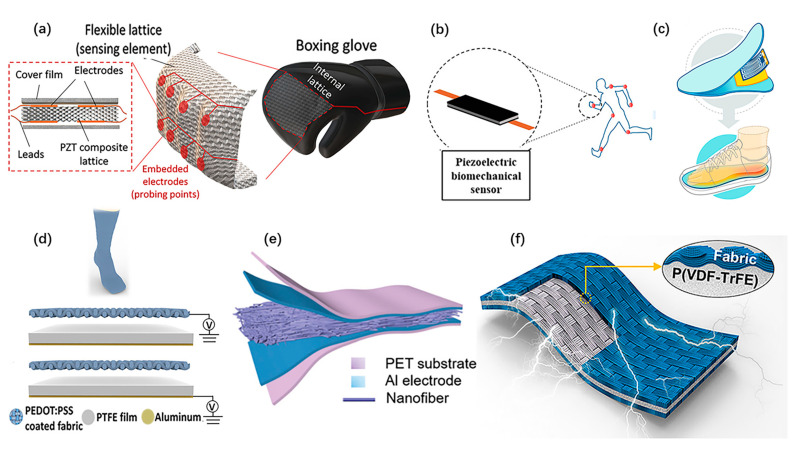
Self-powered wearable systems based on PENG. (**a**) A PENG arrays integrated on a boxing glove for smart sports. Reproduced with permission from Ref. [37]. Copyright © 2019, John Wiley and Sons. (**b**) A highly stretchable piezoelectric biomechanical sensor. Reproduced with permission from Ref. [80]. Copyright © 2020, American Chemical Society. (**c**) PENG adapted to drive a commercial electric heating sheet. Reproduced with permission from Ref. [48]. Copyright © 2020, American Chemical Society. (**d**) A self-powered and self-functional sock based on hybrid nanogenerators. Reproduced with permission from Ref. [32]. Copyright © 2019, American Chemical Society. (**e**) A muscle-fiber-inspired nonwoven piezoelectric textile for health monitoring. Reproduced with permission from Ref. [52]. Copyright © 2020, Wiley-VCH. (**f**) A highly flexible fabric-based wearable PENG. Reproduced with permission from Ref. [72]. Copyright © 2020, Elsevier.

Wearable PENGs demonstrate potential applications for power supply, motion monitoring and health monitoring in wearable systems as well. Desheng Yao et al. presented a wearable boxing glove based on 3D printed flexible piezoelectric lattice with stretch dominated microarchitectures [37], as shown in Figure 7a. It achieves high electromechanical sensitivity and structural functionality. Spatially resolved and time-resolved mapping of reaction punching forces exerted to knuckles of the hand during boxing activities could be obtained. Iqra Choudhry et al. reported a nanocomposite-based PENG fabricated by dispersing various piezoelectric nanoparticles (BaTiO_3_, ZnO, and PZT) graphene nano-powder in a silicone matrix [80]. As shown in Figure 7b, it serves as a biomechanical energy harvester and a self-powered motion sensor. Sun Yue et al. proposed a ZnO/PAN nanofiber-based PENG integrated with a plate heater for personal thermal management [48], as shown in Figure 7c. Minglu Zhu et al. designed a self-sufficient sock composed of hybrid nanogenerators [32], as shown in Figure 7d. It shows good ability in energy harvesting and motion sensing. Yuanjie Sun et al. proposed a muscle-fiber-inspired nonwoven piezoelectric textile with tunable mechanical properties to mimic the muscle fiber, as shown in Figure 7e [52]. It achieves high sensitivity in the monitoring of various physiological signals. Jaegyu Kim et al. developed a highly flexible fabric-based wearable PENG with high efficiency and strong integration [72], as shown in Figure 7f. Beyond these, Shu Du et al. developed a bio-inspired hybrid patch with a PENG embedded [79]. The PENG is employed as an electrical stimulator to facilitate skin wound healing.

## 4. Self-Powered Implantable Systems

### 4.1. Self-Powered Implantable Systems Based on TENG

**Figure 8:**
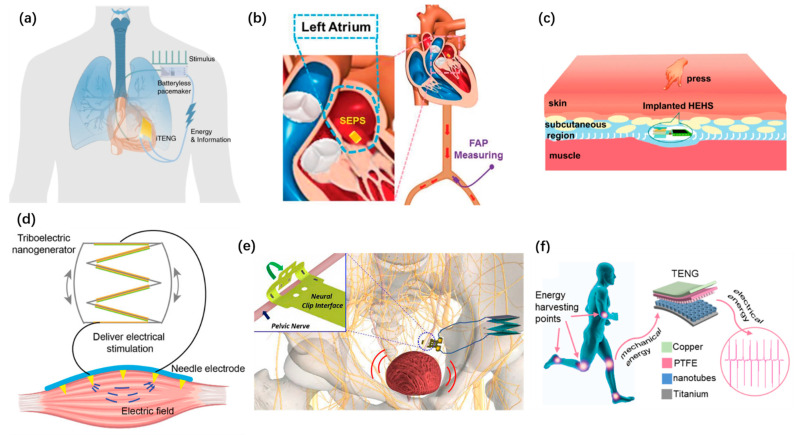
Self-powered implantable systems based on TENG. (**a**) A symbiotic cardiac pacemaker powered by TENG. Reproduced with permission from Ref. [101]. Copyright © 2019, Springer Nature. (**b**) A self-powered endocardial pressure sensor. Reproduced with permission from Ref. [105]. Copyright © 2018, Wiley-VCH. (**c**) An implanted hybrid energy harvesting system. Reproduced with permission from Ref. [92]. Copyright © 2020, Springer Nature. (**d**) Electrical muscle stimulation directly powered by TENG. Reproduced with permission from Ref. [98]. Copyright © 2019, Wiley-VCH. (**e**) A TENG neurostimulator integrated with neural clip interface. Reproduced with permission from Ref. [108]. Copyright © 2019, Elsevier. (**f**) A self-powered treatment to charge titanium implant surface. Reproduced with permission from Ref. [99]. Copyright © 2020, Elsevier.

Harvesting energy from the biomechanical energy of heartbeats, blood pressure, and other biological rhythms to power implantable electronic devices has had an upsurge in recent years. Han Ouyang et al. developed an implanted symbiotic cardiac pacemaker based on TENG [101], as shown in Figure 8a. The implanted TENG can obtain 0.495 μJ electrical energy in each cardiac cycle. Liu Zhuo et al. reported a self-powered endocardial pressure sensor using TENG [105], as shown in Figure 8b. It can monitor in real time to detect arrhythmias. Owing to its specialty in lightweight and flexibility, TENG can be implanted in subcutaneous tissues. Hu Li et al. proposed a hybrid energy harvesting system that consisted of a TENG and a glucose fuel cell [92], as shown in Figure 8c. This design strengthened the flexibility of harvesting multiple sources of bioenergies and enhanced electrical outputs. The high-voltage outputs of TENG were adopted to stimulate muscles and nerves as well. Jiahui Wang et al. proposed a self-powered muscle stimulation system based on TENG [98], as shown in Figure 8d. The TENG can directly stimulate the muscle to treat the muscle dysfunction. The multi-channel electrode adheres well to the surface of muscle. Sanghoon Lee et al. proposed a TENG neurostimulator to realize the mechano-neuromodulation of autonomic pelvic nerves [108]. As shown in Figure 8e, the stimulator system consists of a stacked TENG and a flexible neural clip interface. Rui Shi et al. proposed a self-powered treatment strategy employing a TENG to charge a titanium implant surface [99], as shown in Figure 8f. The charged titanium implant shows a suitable antibacterial property. It can serve as an antibacterial biofilm and helps to promote the osseointegration.

### 4.2. Self-Powered Implantable Systems Based on PENG

**Figure 9:**
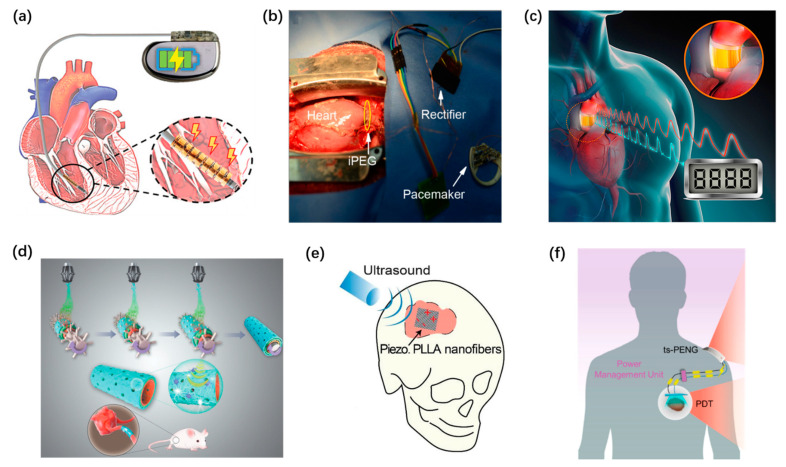
Self-powered implantable systems based on PENG. (**a**) A kirigami inspired PENG. Reproduced with permission from Ref. [97]. Copyright © 2021, Wiley-VCH. (**b**) A self-powered leadless cardiac pacemaker. Reproduced with permission from Ref. [106]. Copyright © 2019, American Chemical Society. (**c**) PENG for in vivo blood pressure monitoring. Reproduced with permission from Ref. [119]. Copyright © 2016, Elsevier. (**d**) A PENG scaffold for tissue healing. Reproduced with permission from Ref. [100]. Copyright © 2020, Wiley-VCH. (**e**) A battery-less electrical stimulator serving as a bone scaffold. Reproduced with permission from Ref. [85]. Copyright © 2020, Elsevier. (**f**) A self-powered photodynamic therapy system. Reproduced with permission from Ref. [104]. Copyright © 2020, American Chemical Society.

Implantable PENGs and piezoelectric sensors have shown great potential in the evaluation and diagnosis of cardiovascular diseases. Great efforts have been made to power cardiac pacemakers by using PENGs. Zhe Xu et al. developed a kirigami inspired PENG [97], as shown in Figure 9a. The PENG is fixed on the lead of the pacemaker to harvest energy from the lead’s motion caused by heartbeats. Zhiran Yi et al. proposed a self-powered leadless cardiac pacemaker [96]. The PENG used to power the pacemaker obtains a short-circuit current of 30 μA and an open-circuit voltage of 8.1 V. Li Ning et al. proposed an implantable PENG, as shown in Figure 9b. It can directly power a cardiac pacemaker via a rectifier [106]. Xiaoliang cheng et al. presented an implantable self-powered blood pressure monitor based on a PENG [119], as shown in Figure 9c. Good linearity was achieved between the peak output voltage of the PENG and the flow pressure, with a sensitivity of 173 mV/mmHg. As shown in Figure 9d, Qian Yun et al. designed a ZnO based PENG scaffold [100]. It plays a role as an in vivo stimulus to accelerate the speed of tissue healing and nerve conducting. Ritopa Das et al. proposed a new method for bone regeneration [85], as shown in Figure 9e. A biodegradable PENG scaffold driven by ultrasound is adapted as an electrical stimulator to promote bone regeneration. Liu Zhuo and others designed a PENG to power the photodynamic therapy system for cancer treatment to inhibit the growth of subcutaneous tumor cells in mice [104], as shown in Figure 9f. The inhibition rate reached 87.46%.

## 5. Conclusions

This article has reviewed the recent developments of self-powered systems based on TENG and PENG for wearable and implantable applications. The materials and structures for nanogenerators and their wearable and implantable applications are discussed. In terms of materials, biodegradable PVA, PLLA, silk, and so on are introduced to increase the possibility of implantation. Additionally, liquid crystal materials and hydrogel materials are used to increase the tensile strength and affinity. As to the device structures, three-dimensional structures, textile structures, and spring-mass structures of hybrid nanogenerators show good performance in wearable applications. In addition, various thin-film structures with well-designed transformation or separation are valuable for in vivo energy harvesting as the application of self-powered wearable systems. They can be directly attached to the skin and worn as part of the clothing or accessory for motion monitoring, health monitoring, wound repairing, etc. Implantable systems are supposed to have the characteristics of good biocompatibility and high durability. So far, the applications on the battery-less cardiac pacemaker, in vivo health monitoring, in vivo stimulation, and therapy are promising.

Considering the abovementioned progress on wearable and implantable self-powered systems, it is still at its infancy stage of development. Triboelectric and piezoelectric materials with high charge density, good biocompatibility and ease of manufacture will be crucial. To realize fully self-powered wearable and implantable systems, power management circuits with high efficiency [121,122] would be indispensable for nanogenerators.

## Figures and Tables

**Table 1:** Wearable and implantable TENGs and PENGs.

Nanogenerator Type	Wearable TENGs	Wearable PENGs	Implantable TENGs	Implantable PENGs
Location of Installation	chest [78]	knee [80,108]; foot [32,48,77]	heart and pericardium [87,101,105]	pacemaker lead [97,116]
	elbow [26,56]	chest [29,50];	duodenum [120];	stomach [103]
	knee [26,30,56]	neck [18,19,29,50,52]	tumor cells [94]	lung [86]
	waist [21,25,27,45,55,74,81]	elbow [49,72,76]	the surface of bone [99]	heart [83,84,86,106,110,114]
	eye [23,28]	wrist [18,29,50]	the subdermal dorsal region [93]	blood vessel [29,112,119]
	ear [20]; foot [42,56,69]; skin [17,78]	hand [18,37,76]	skin underneath [88,92]	skin underneath [104]
	hand [26,33,38,44,53,65]	skin [79]		
Biomechanical Energy Source	walking, running [42,53,56,69,80]	walking, running [32,48,77]	joint movement [99]	motions of leg [104]
	stretching [21,25,26,33,53,56]	stretching [29,72] [76,79]	blood pressure [93]	blood pressure [112,119]
	blinking [23,28]	joint movement [49,50,81]	the peristalsis of duodenum [120]	motions of stomach [103]
	shake and pat [17,38,43,82]	breathing [18]	heartbeat [87,93,101,105]	heartbeat [83,106,110,114,116]
	motions of finger [44,65]	pulse [19,52]	breathing [88,92,93]	breathing [29]
	breathing [21,27]; pulse [45]	punching [37]		motions of heart lead [84,86,97,115]
	speaking [20]; touching [55,74,78]			
Materials	PTFE [17,27,42,55]	ZnO [48,81]	PTFE [87,94,101,105]	PVDF [29,83,104,119]
	Kapton [20,45]; PVDF [33,65]	PVDF [29,49,77,79]	PLGA [88,93]	ZnO [84]
	Nylon [24,27,74,78]	P(VDF-TrFE) [18,72,78]	PDMS [87,120]	PVDF-TrFE [97,114,116]
	Mxene [56]; carbon nanotube [44,53]	Dopamine [29]; PMN-PT [19,77]	PVA [93]	PZT [86,103]
	hydrogel [23,25]	balsa wood [50]; PZT [32,37,80]	PET [87,92]	PMN-PT [106,110]
	Ecoflex [55,56]; liquid metal [38]	BaTiO3 [80]	Kapton [87,92,101]	
	rubber [21,38]; silicone [25,30]		titanium [92,94,99,101]	
	PVA [25,45]; silk [53];			
Applications	human-machine interface [20,55]	motion monitoring [19,29,37,50]	anti-bacteria [99]	in vivo health monitoring [83,97,119]
	motion monitoring [21,27,33,56,74]	health monitoring [18,49,52]	anti-tumor therapy [94]	in vivo therapying [29,103,104,114]
	health monitoring [27,45,78]	wound healing [79]	in vivo health monitoring [101,105,120]	regeneration of tissues [85]
	eye motion monitoring [23,28]	power supply [18,32,72,77]	electrical stimulator [98]	implanted sensor [112]
	voice and gesture recognition [20,44,65]		power supply [87,88,92,93]	power supply [84,106,110]
	drug delivery [17]			
	power supply [30,38,65,69,81]			

## References

[1] Chan M., Estève D., Fourniols J.-Y., Escriba C., Campo E. (2012). Smart wearable systems: Current status and future challenges. Artif. Intell. Med..

[2] Zeng W., Shu L., Li Q., Chen S., Wang F., Tao X.-M. (2014). Fiber-Based Wearable Electronics: A Review of Materials, Fabrication, Devices, and Applications. Adv. Mater..

[3] Gao M., Wang P., Jiang L., Wang B., Yao Y., Liu S., Chu D., Cheng W., Lu Y. (2021). Power generation for wearable systems. Energy Environ. Sci..

[4] Jiang D.J., Shi B.J., Ouyang H., Fan Y.B., Wang Z.L., Li Z. (2020). Emerging Implantable Energy Harvesters and Self-Powered Implantable Medical Electronics. ACS Nano.

[5] Xue R.-F., Cheng K.-W., Je M. (2012). High-Efficiency Wireless Power Transfer for Biomedical Implants by Optimal Resonant Load Transformation. IEEE Trans. Circuits Syst. I Regul. Pap..

[6] Kim B.J., Kim D.H., Lee Y.-Y., Shin H.-W., Han G.S., Hong J.S., Mahmood K., Ahn T.K., Joo Y.-C., Hong K.S. (2015). Highly efficient and bending durable perovskite solar cells: Toward a wearable power source. Energy Environ. Sci..

[7] Leonov V., Vullers R.J.M. (2009). Wearable electronics self-powered by using human body heat: The state of the art and the perspective. J. Renew. Sustain. Energy.

[8] Wang Z.L., Song J.H. (2006). Piezoelectric nanogenerators based on zinc oxide nanowire arrays. Science.

[9] Wang X.D., Song J.H., Liu J., Wang Z.L. (2007). Direct-Current Nanogenerator Driven by Ultrasonic Waves. Science.

[10] Fan F.-R., Tian Z.-Q., Wang Z.L. (2012). Flexible triboelectric generator. Nano Energy.

[11] Fan X., Chen J., Yang J., Bai P., Li Z.L., Wang Z.L. (2015). Ultrathin, Rollable, Paper-Based Triboelectric Nanogenerator for Acoustic Energy Harvesting and Self-Powered Sound Recording. ACS Nano.

[12] Zhu G., Zhou Y.S., Bai P., Meng X.S., Jing Q.S., Chen J., Wang Z.L. (2014). A Shape-Adaptive Thin-Film-Based Approach for 50% High-Efficiency Energy Generation Through Micro-Grating Sliding Electrification. Adv. Mater..

[13] Wu W., Bai S., Yuan M., Qin Y., Wang Z.L., Jing T. (2012). Lead Zirconate Titanate Nanowire Textile Nanogenerator for Wearable Energy-Harvesting and Self-Powered Devices. ACS Nano.

[14] Seung W., Gupta M.K., Lee K.Y., Shin K.-S., Lee J.-H., Kim T.Y., Kim S., Lin J., Kim J.H., Kim S.-W. (2015). Nanopatterned Textile-Based Wearable Triboelectric Nanogenerator. ACS Nano.

[15] Lee J.-H., Lee K.Y., Gupta M.K., Kim T.Y., Lee D.-Y., Oh J., Ryu C., Yoo W.J., Kang C.-Y., Yoon S.-J. (2014). Highly Stretchable Piezoelectric-Pyroelectric Hybrid Nanogenerator. Adv. Mater..

[16] Fan F.R., Tang W., Wang Z.L. (2016). Flexible Nanogenerators for Energy Harvesting and Self-Powered Electronics. Adv. Mater..

[17] Jiang Q., Wu C.S., Wang Z.J., Wang A.C., He J.H., Wang Z.L., Alshareef H.N. (2018). MXene electrochemical microsupercapacitor integrated with triboelectric nanogenerator as a wearable self-charging power unit. Nano Energy.

[18] Wang A.C., Hu M., Zhou L.W., Qiang X.Y. (2018). Self-Powered Wearable Pressure Sensors with Enhanced Piezoelectric Properties of Aligned P(VDF-TrFE)/MWCNT Composites for Monitoring Human Physiological and Muscle Motion Signs. Nanomaterials.

[19] Jung Y.H., Hong S.K., Wang H.S., Han J.H., Pham T.X., Park H., Kim J., Kang S., Yoo C.D., Lee K.J. (2020). Flexible Piezoelectric Acoustic Sensors and Machine Learning for Speech Processing. Adv. Mater..

[20] Guo H.Y., Pu X.J., Chen J., Meng Y., Yeh M.-H., Liu G.L., Tang Q., Chen B.D., Liu D., Qi S. (2018). A highly sensitive, self-powered triboelectric auditory sensor for social robotics and hearing aids. Sci. Robot..

[21] Yi F., Lin L., Niu S.M., Yang P.K., Wang Z.N., Chen J., Zhou Y.S., Zi Y.L., Wang J., Liao Q.L. (2015). Stretchable-Rubber-Based Triboelectric Nanogenerator and Its Application as Self-Powered Body Motion Sensors. Adv. Funct. Mater..

[22] Ning C., Dong K., Cheng R.W., Yi J., Ye C.Y., Peng X., Sheng F.F., Jiang Y., Wang Z.L. (2021). Flexible and Stretchable Fiber-Shaped Triboelectric Nanogenerators for Biomechanical Monitoring and Human-Interactive Sensing. Adv. Funct. Mater..

[23] Lu X., Zheng L., Zhang H.D., Wang W.H., Wang Z.L., Sun C.W. (2020). Stretchable, transparent triboelectric nanogenerator as a highly sensitive self-powered sensor for driver fatigue and distraction monitoring. Nano Energy.

[24] Chen H.M., Bai L., Li T., Zhao C., Zhang J.S., Zhang N., Song G.F., Gan Q.Q., Xu Y. (2018). Wearable and robust triboelectric nanogenerator based on crumpled gold films. Nano Energy.

[25] Guan Q.B., Lin G.H., Gong Y.Z., Wang J.F., Tan W.Y., Bao D.Q., Liu Y.N., You Z.W., Sun X.H., Wen Z. (2019). Highly efficient self-healable and dual responsive hydrogel-based deformable triboelectric nanogenerators for wearable electronics. J. Mater. Chem. A.

[26] Gogurla N., Roy B., Park J.-Y., Kim S. (2019). Skin-contact actuated single-electrode protein triboelectric nanogenerator and strain sensor for biomechanical energy harvesting and motion sensing. Nano Energy.

[27] Zhang H., Zhang J.W., Hu Z.W., Quan L.W., Shi L., Chen J.K., Xuan W.P., Zhang Z.C., Dong S.R., Luo J.K. (2019). Waist-wearable wireless respiration sensor based on triboelectric effect. Nano Energy.

[28] Pu X.J., Guo H.Y., Chen J., Wang X., Xi Y., Hu C.G., Wang Z.L. (2017). Eye motion triggered self-powered mechnosensational communication system using triboelectric nanogenerator. Sci. Adv..

[29] Li T., Qu M.H., Carlos O.R., Gu L., Jin F., Yuan T., Wu X.W., Xiao J.J., Wang T., Dong W. (2021). High-Performance Poly(vinylidene difluoride)/Dopamine Core/Shell Piezoelectric Nanofiber and Its Application for Biomedical Sensors. Adv. Mater..

[30] Zou Y., Tan P.C., Shi B.J., Ouyang H., Jiang D.J., Liu Z., Li H., Yu M., Wang C., Qu X.C. (2019). A bionic stretchable nanogenerator for underwater sensing and energy harvesting. Nat. Commun..

[31] Zhu M.L., Sun Z.D., Zhang Z.X., Shi Q.F., He T.Y.Y., Liu H.C., Chen T., Lee C.K. (2020). Haptic-feedback smart glove as a creative human-machine interface (HMI) for virtual/augmented reality applications. Sci. Adv..

[32] Zhu M.L., Shi Q.F., He T.Y.Y., Yi Z.R., Ma Y.M., Yang B., Chen T., Lee C. (2019). Self-Powered and Self-Functional Cotton Sock Using Piezoelectric and Triboelectric Hybrid Mechanism for Healthcare and Sports Monitoring. ACS Nano.

[33] Zhou Z.H., Padgett S., Cai Z.X., Conta G., Wu Y.F., He Q., Zhang S.L., Sun C.C., Liu J., Fan E.D. (2020). Single-layered ultra-soft washable smart textiles for all-around ballistocardiograph, respiration, and posture monitoring during sleep. Biosens. Bioelectron..

[34] Zhou T., Zhang C., Han C.B., Fan F.R., Tang W., Wang Z.L. (2014). Woven Structured Triboelectric Nanogenerator for Wearable Devices. ACS Appl. Mater. Interfaces.

[35] Zhang W., Yang H.M., Li L., Lin S.Q., Ji P.Y., Hu C.G., Zhang D.Z., Xi Y. (2020). Flexible piezoelectric nanogenerators based on a CdS nanowall for self-powered sensors. Nanotechnology.

[36] Wang J., Li S., Yi F., Zi Y., Lin J., Wang X., Xu Y., Wang Z.L. (2016). Sustainably powering wearable electronics solely by biomechanical energy. Nat. Commun..

[37] Yao D.S., Cui H.C., Hensleigh R., Smith P., Alford S., Bernero D., Bush S., Mann K., Wu H.F., Chin-Nieh M. (2019). Achieving the Upper Bound of Piezoelectric Response in Tunable, Wearable 3D Printed Nanocomposites. Adv. Funct. Mater..

[38] Yang Y.Q., Sun N., Wen Z., Cheng P., Zheng H.C., Shao H.Y., Xia Y.J., Chen C., Lan H.W., Xie X.K. (2018). Liquid-Metal-Based Super-Stretchable and Structure-Designable Triboelectric Nanogenerator for Wearable Electronics. ACS Nano.

[39] Yang Y., Pan H., Xie G.Z., Jiang Y.D., Chen C.X., Su Y.J., Wang Y., Tai H.L. (2020). Flexible piezoelectric pressure sensor based on polydopamine-modified BaTiO3/PVDF composite film for human motion monitoring. Sens. Actuators A Phys..

[40] Yang L., Zhao Q.Y., Chen K.N., Ma Y.Z., Wu Y.P., Ji H.L., Qiu J.H. (2020). PVDF-Based Composition-Gradient Multilayered Nanocomposites for Flexible High-Performance Piezoelectric Nanogenerators. ACS Appl. Mater. Interfaces.

[41] Yan J., Liu M., Jeong Y.G., Kang W.M., Li L., Zhao Y.X., Deng N.P., Cheng B.W., Yang G. (2019). Performance enhancements in poly(vinylidene fluoride)-based piezoelectric nanogenerators for efficient energy harvesting. Nano Energy.

[42] Yan C., Gao Y.Y., Zhao S.L., Zhang S.L., Zhou Y.H., Deng W.L., Li Z.W., Jiang G., Jin L., Tian G. (2020). A linear-to-rotary hybrid nanogenerator for high-performance wearable biomechanical energy harvesting. Nano Energy.

[43] Xin Y., Qi X.H., Qian C.H., Tian H.Y., Ling Z.B., Jiang Z.J. (2014). A Wearable Respiration and Pulse Monitoring System Based on PVDF Piezoelectric Film. Integr. Ferroelectr..

[44] Wen F., Sun Z., He T., Shi Q., Zhu M., Zhang Z., Li L., Zhang T., Lee C. (2020). Machine Learning Glove Using Self-Powered Conductive Superhydrophobic Triboelectric Textile for Gesture Recognition in VR/AR Applications. Adv. Sci..

[45] Wang R.X., Mu L.W., Bao Y.K., Lin H., Ji T., Shi Y.J., Zhu J.H., Wu W.Z. (2020). Holistically Engineered Polymer–Polymer and Polymer–Ion Interactions in Biocompatible Polyvinyl Alcohol Blends for High-Performance Triboelectric Devices in Self-Powered Wearable Cardiovascular Monitorings. Adv. Mater..

[46] Wang J., Li X.H., Zi Y.L., Wang S.H., Li Z.L., Zheng L., Yi F., Li S.M., Wang Z.L. (2015). A Flexible Fiber-Based Supercapacitor-Triboelectric-Nanogenerator Power System for Wearable Electronics. Adv. Mater..

[47] Viola G., Chang J.K., Maltby T., Steckler F., Jomaa M., Sun J.F., Edusei J., Zhang D., Vilches A., Gao S. (2020). Bioinspired Multiresonant Acoustic Devices Based on Electrospun Piezoelectric Polymeric Nanofibers. ACS Appl. Mater. Interfaces.

[48] Sun Y., Liu Y., Zheng Y.D., Li Z.J., Fan J., Wang L., Liu X.Q., Liu J., Shou W. (2020). Enhanced Energy Harvesting Ability of ZnO/PAN Hybrid Piezoelectric Nanogenerators. ACS Appl. Mater. Interfaces.

[49] Ahn S., Cho Y., Park S., Kim J., Sun J., Ahn D., Lee M., Kim D., Kim T., Shin H. (2020). Wearable multimode sensors with amplified piezoelectricity due to the multi local strain using 3D textile structure for detecting human body signals. Nano Energy.

[50] Sun J., Guo H.Y., Ribera J., Wu C.S., Tu K.K., Binelli M., Panzarasa G., Schwarze F.W.M.R., Wang Z.L., Burgert I. (2020). Sustainable and Biodegradable Wood Sponge Piezoelectric Nanogenerator for Sensing and Energy Harvesting Applications. ACS Nano.

[51] Su Y.J., Wang J.J., Wang B., Yang T.N., Yang B.X., Xie G.Z., Zhou Y.H., Zhang S.L., Tai H.L., Cai Z.X. (2020). Alveolus-Inspired Active Membrane Sensors for Self-Powered Wearable Chemical Sensing and Breath Analysis. ACS Nano.

[52] Su Y.J., Chen C.X., Pan H., Yang Y., Chen G.R., Zhao X., Li W.X., Gong Q.C., Xie G.Z., Zhou Y.H. (2021). Muscle Fibers Inspired High-Performance Piezoelectric Textiles for Wearable Physiological Monitoring. Adv. Funct. Mater..

[53] Su M., Brugger J., Kim B. (2020). Simply Structured Wearable Triboelectric Nanogenerator Based on a Hybrid Composition of Carbon Nanotubes and Polymer Layer. Int. J. Precis. Eng. Manuf. Technol..

[54] Song Y., Min J.H., Yu Y., Wang H.B., Yang Y.R., Zhang H.X., Gao W. (2020). Wireless battery-free wearable sweat sensor powered by human motion. Sci. Adv..

[55] Shi Q.F., Lee C.K. (2019). Self-Powered Bio-Inspired Spider-Net-Coding Interface Using Single-Electrode Triboelectric Nanogenerator. Adv. Sci..

[56] Salauddin M., Rana S.M.S., Sharifuzzaman M., Rahman M.T., Park C., Cho H., Maharjan P., Bhatta T., Park J.Y. (2021). A Novel MXene/Ecoflex Nanocomposite-Coated Fabric as a Highly Negative and Stable Friction Layer for High-Output Triboelectric Nanogenerators. Adv. Energy Mater..

[57] Pu X., Liu M.M., Chen X.Y., Sun J.M., Du C.H., Zhang Y., Zhai J.Y., Hu W.G., Wang Z.L. (2017). Ultrastretchable, transparent triboelectric nanogenerator as electronic skin for biomechanical energy harvesting and tactile sensing. Sci. Adv..

[58] Peng X., Dong K., Ye C.Y., Jiang Y., Zhai S.Y., Cheng R.W., Liu D., Gao X.P., Wang J., Wang Z.L. (2020). A breathable, biodegradable, antibacterial, and self-powered electronic skin based on all-nanofiber triboelectric nanogenerators. Sci. Adv..

[59] Park C., Koo M., Song G., Cho S.M., Kang H.S., Park T.H., Kim E.H., Park C. (2020). Surface-Conformal Triboelectric Nanopores via Supramolecular Ternary Polymer Assembly. ACS Nano.

[60] Pan C.F., Liu D.Y., Ford M.J., Majidi C. (2020). Ultrastretchable, Wearable Triboelectric Nanogenerator Based on Sedimented Liquid Metal Elastomer Composite. Adv. Mater. Technol..

[61] Niu Q.Q., Huang L., Lv S.S., Shao H.L., Fan S.N., Zhang Y.P. (2020). Pulse-driven bio-triboelectric nanogenerator based on silk nanoribbons. Nano Energy.

[62] Mitcheson P.D., Yeatman E.M., Rao G.K., Holmes A.S., Green T.C. (2008). Energy Harvesting from Human and Machine Motion for Wireless Electronic Devices. Proc. IEEE.

[63] Maity K., Garain S., Henkel K., Schmeißer D., Mandal D. (2020). Self-Powered Human-Health Monitoring through Aligned PVDF Nanofibers Interfaced Skin-Interactive Piezoelectric Sensor. ACS Appl. Polym. Mater..

[64] Maharjan P., Bhatta T., Cho H., Hui X., Park C., Yoon S., Salauddin M., Rahman M.T., Rana S.M.S., Park J.Y. (2020). A Fully Functional Universal Self-Chargeable Power Module for Portable/Wearable Electronics and Self-Powered IoT Applications. Adv. Energy Mater..

[65] Ma L.Y., Zhou M.J., Wu R.H., Patil A., Gong H., Zhu S.H., Wang T.T., Zhang Y.F., Shen S., Dong K. (2020). Continuous and Scalable Manufacture of Hybridized Nano-Micro Triboelectric Yarns for Energy Harvesting and Signal Sensing. ACS Nano.

[66] Lu L.J., Ding W.Q., Liu J.Q., Yang B. (2020). Flexible PVDF based piezoelectric nanogenerators. Nano Energy.

[67] Liu Z.R., Nie J.H., Miao B., Li J.D., Cui Y.B., Wang S., Zhang X.D., Zhao G.R., Deng Y.B., Wu Y.H. (2019). Self-Powered Intracellular Drug Delivery by a Biomechanical Energy-Driven Triboelectric Nanogenerator. Adv. Mater..

[68] Cheng X.L., Meng B., Zhang X.S., Han M.D., Su Z.M., Zhang H.X. (2015). Wearable electrode-free triboelectric generator for harvesting biomechanical energy. Nano Energy.

[69] Lin Z.M., Wu Z.Y., Zhang B.B., Wang Y.C., Guo H.Y., Liu G.L., Chen C.Y., Chen Y.L., Yang J., Wang Z.L. (2019). A Triboelectric Nanogenerator-Based Smart Insole for Multifunctional Gait Monitoring. Adv. Mater. Technol..

[70] Li T., Feng Z.Q., Qu M.H., Yan K., Yuan T., Gao B.B., Wang T., Dong W., Zheng J. (2019). Core/Shell Piezoelectric Nanofibers with Spatial Self-Orientated Beta-Phase Nanocrystals for Real-Time Micropressure Monitoring of Cardiovascular Walls. ACS Nano.

[71] Lee M., Chen C.Y., Wang S., Cha S.N., Park Y.J., Kim J.M., Chou L.J., Wang Z.L. (2012). A Hybrid Piezoelectric Structure for Wearable Nanogenerators. Adv. Mater..

[72] Kim J., Byun S., Lee S., Ryu J., Cho S., Oh C., Kim H., No K., Ryu S., Lee Y.M. (2020). Cost-effective and strongly integrated fabric-based wearable piezoelectric energy harvester. Nano Energy.

[73] Khurana V., Kisannagar R.R., Domala S.S., Gupta D. (2020). In Situ Polarized Ultrathin PVDF Film-Based Flexible Piezoelectric Nanogenerators. ACS Appl. Electron. Mater..

[74] Jiang Y., Dong K., Li X., An J., Wu D.Q., Peng X., Yi J., Ning C., Cheng R.W., Yu P.T. (2021). Stretchable, Washable, and Ultrathin Triboelectric Nanogenerators as Skin-Like Highly Sensitive Self-Powered Haptic Sensors. Adv. Funct. Mater..

[75] Jeong S.-H., Lee Y., Lee M.-G., Song W.J., Park J.-U., Sun J.-Y. (2021). Accelerated wound healing with an ionic patch assisted by a triboelectric nanogenerator. Nano Energy.

[76] Guan X.Y., Xu B.G., Gong J.L. (2020). Hierarchically architected polydopamine modified BaTiO3@P(VDF-TrFE) nanocomposite fiber mats for flexible piezoelectric nanogenerators and self-powered sensors. Nano Energy.

[77] Gu L., Liu J.M., Cui N.Y., Xu Q., Du T., Zhang L., Wang Z., Long C.B., Qin Y. (2020). Enhancing the current density of a piezoelectric nanogenerator using a three-dimensional intercalation electrode. Nat. Commun..

[78] Fan W.J., He Q., Meng K.Y., Tan X.L., Zhou Z.H., Zhang G.Q., Yang J., Wang Z.L. (2020). Machine-knitted washable sensor array textile for precise epidermal physiological signal monitoring. Sci. Adv..

[79] Du S., Zhou N.Y., Gao Y.J., Xie G., Du H.Y., Jiang H., Zhang L.B., Tao J., Zhu J.T. (2020). Bioinspired hybrid patches with self-adhesive hydrogel and piezoelectric nanogenerator for promoting skin wound healing. Nano Res..

[80] Choudhry I., Khalid H.R., Lee H.-K. (2020). Flexible Piezoelectric Transducers for Energy Harvesting and Sensing from Human Kinematics. ACS Appl. Electron. Mater..

[81] Chen C.Y., Guo H.Y., Chen L.J., Wang Y.C., Pu X.J., Yu W.D., Wang F.M., Du Z.Q., Wang Z.L. (2020). Direct Current Fabric Triboelectric Nanogenerator for Biomotion Energy Harvesting. ACS Nano.

[82] Cao R., Pu X.J., Du X.Y., Yang W., Wang J.N., Guo H.Y., Zhao S.Y., Yuan Z.Q., Zhang C., Li C.J. (2018). Screen-Printed Washable Electronic Textiles as Self-Powered Touch/Gesture Tribo-Sensors for Intelligent Human–Machine Interaction. ACS Nano.

[83] Sun R.J., Carreira S.C., Chen Y., Xiang C.Q., Xu L.L., Zhang B., Chen M.D., Farrow I., Scarpa F., Rossiter J. (2019). Stretchable Piezoelectric Sensing Systems for Self-Powered and Wireless Health Monitoring. Adv. Mater. Technol..

[84] Jin C.R., Dong L., Xu Z., Closson A., Cabe A., Gruslova A., Jenney S., Escobedo D., Elliott J., Zhang M. (2021). Skin-like Elastomer Embedded Zinc Oxide Nanoarrays for Biomechanical Energy Harvesting. Adv. Mater. Interfaces.

[85] Das R., Curry E.J., Le T.T., Awale G., Liu Y., Li S.Y., Contreras J., Bednarz C., Millender J., Xin X. (2020). Biodegradable nanofiber bone-tissue scaffold as remotely-controlled and self-powering electrical stimulator. Nano Energy.

[86] Dagdeviren C., Shi Y., Joe P., Ghaffari R., Balooch G., Usgaonkar K., Gur O., Tran P.L., Crosby J.R., Meyer M. (2015). Conformal piezoelectric systems for clinical and experimental characterization of soft tissue biomechanics. Nat. Mater..

[87] Zheng Q., Zhang H., Shi B.J., Xue X., Liu Z., Jin Y.M., Ma Y., Zou Y., Wang X.X., An Z. (2016). In Vivo Self-Powered Wireless Cardiac Monitoring via Implantable Triboelectric Nanogenerator. ACS Nano.

[88] Li Z., Feng H.Q., Zheng Q., Li H., Zhao C.C., Ouyang H., Noreen S., Yu M., Su F., Liu R.P. (2018). Photothermally tunable biodegradation of implantable triboelectric nanogenerators for tissue repairing. Nano Energy.

[89] Tian J.J., Shi R., Liu Z., Ouyang H., Yu M., Zhao C.C., Zou Y., Jiang D.J., Zhang J.S., Li Z. (2019). Self-powered implantable electrical stimulator for osteoblasts’ proliferation and differentiation. Nano Energy.

[90] Zheng Q., Shi B.J., Fan F.R., Wang X.X., Yan L., Yuan W.W., Wang S.H., Liu H., Li Z., Wang Z.L. (2014). In Vivo Powering of Pacemaker by Breathing-Driven Implanted Triboelectric Nanogenerator. Adv. Mater..

[91] Yang J., Chen J., Su Y.J., Jing Q.S., Li Z.L., Yi F., Wen X.N., Wang Z.N., Wang Z.L. (2015). Eardrum-Inspired Active Sensors for Self-Powered Cardiovascular System Characterization and Throat-Attached Anti-Interference Voice Recognition. Adv. Mater..

[92] Li H., Zhang X., Zhao L.M., Jiang D.J., Xu L.L., Liu Z., Wu Y.X., Hu K., Zhang M.R., Wang J.X. (2020). A Hybrid Biofuel and Triboelectric Nanogenerator for Bioenergy Harvesting. Nano Micro Lett..

[93] Zheng Q., Zou Y., Zhang Y.L., Liu Z., Shi B.J., Wang X.X., Jin Y.M., Ouyang H., Li Z., Wang Z.L. (2016). Biodegradable triboelectric nanogenerator as a life-time designed implantable power source. Sci. Adv..

[94] Zhao C.C., Feng H.Q., Zhang L.J., Li Z., Zou Y., Tan P.C., Ouyang H., Jiang D.J., Yu M., Wang C. (2019). Highly Efficient In Vivo Cancer Therapy by an Implantable Magnet Triboelectric Nanogenerator. Adv. Funct. Mater..

[95] Zhang X.-S., Han M.-D., Wang R.-X., Zhu F.-Y., Li Z.-H., Wang W., Zhang H.X. (2013). Frequency-Multiplication High-Output Triboelectric Nanogenerator for Sustainably Powering Biomedical Microsystems. Nano Lett..

[96] Yi Z.R., Xie F., Tian Y.W., Li N., Dong X.X., Ma Y., Huang Y., Hu Y.L., Xu X.B., Qu D. (2020). A Battery- and Leadless Heart-Worn Pacemaker Strategy. Adv. Funct. Mater..

[97] Xu Z., Jin C.R., Cabe A., Escobedo D., Gruslova A., Jenney S., Closson A.B., Dong L., Chen Z., Feldman M.D. (2021). Implantable Cardiac Kirigami-Inspired Lead-Based Energy Harvester Fabricated by Enhanced Piezoelectric Composite Film. Adv. Heal. Mater..

[98] Wang J.H., Wang H., He T.Y.Y., He B.R., Thakor N.V., Lee C. (2019). Investigation of Low-Current Direct Stimulation for Rehabilitation Treatment Related to Muscle Function Loss Using Self-Powered TENG System. Adv. Sci..

[99] Shi R., Zhang J.S., Tian J.J., Zhao C.C., Li Z., Zhang Y.Z., Li Y.S., Wu C.G., Tian W., Li Z. (2020). An effective self-powered strategy to endow titanium implant surface with associated activity of anti-biofilm and osteogenesis. Nano Energy.

[100] Qian Y., Cheng Y., Song J.L., Xu Y., Yuan W.E., Fan C.Y., Zheng X.Y. (2020). Mechano-Informed Biomimetic Polymer Scaffolds by Incorporating Self-Powered Zinc Oxide Nanogenerators Enhance Motor Recovery and Neural Function. Small.

[101] Ouyang H., Liu Z., Li N., Shi B.J., Zou Y., Xie F., Ma Y., Li Z., Li H., Zheng Q. (2019). Symbiotic cardiac pacemaker. Nat. Commun..

[102] Song P., Kuang S., Panwar N., Yang G., Tng D.J.H., Tjin S.C., Ng W.J., Majid M.B.A., Zhu G., Yong K.-T. (2017). A Self-Powered Implantable Drug-Delivery System Using Biokinetic Energy. Adv. Mater..

[103] Lu H.J., Hong Y., Yang Y.Y., Yang Z.B., Shen Y.J. (2020). Battery-Less Soft Millirobot That Can Move, Sense, and Communicate Remotely by Coupling the Magnetic and Piezoelectric Effects. Adv. Sci..

[104] Liu Z., Xu L.L., Zheng Q., Kang Y., Shi B.J., Jiang D.J., Li H., Qu X.C., Fan Y.B., Wang Z.L. (2020). Human Motion Driven Self-Powered Photodynamic System for Long-Term Autonomous Cancer Therapy. ACS Nano.

[105] Liu Z., Ma Y., Ouyang H., Shi B.J., Li N., Jiang D.J., Xie F., Qu D., Zou Y., Huang Y. (2019). Transcatheter Self-Powered Ultrasensitive Endocardial Pressure Sensor. Adv. Funct. Mater..

[106] Li N., Yi Z.R., Ma Y., Xie F., Huang Y., Tian Y.W., Dong X.X., Liu Y., Shao X., Jin L. (2019). Direct Powering a Real Cardiac Pacemaker by Natural Energy of a Heartbeat. ACS Nano.

[107] Lee T.I., Lee S., Lee E., Sohn S., Lee Y., Lee S., Moon G., Kim D., Kim Y.S., Myoung J.M. (2013). High-Power Density Piezoelectric Energy Harvesting Using Radially Strained Ultrathin Trigonal Tellurium Nanowire Assembly. Adv. Mater..

[108] Lee S., Wang H., Peh W.Y.X., He T.Y.Y., Yen S.-C., Thakor N.V., Lee C. (2019). Mechano-neuromodulation of autonomic pelvic nerve for underactive bladder: A triboelectric neurostimulator integrated with flexible neural clip interface. Nano Energy.

[109] Kim D.H., Shin H.J., Lee H., Jeong C.K., Park H., Hwang G.-T., Lee H.-Y., Joe D.J., Han J.H., Lee S.H. (2017). In Vivo Self-Powered Wireless Transmission Using Biocompatible Flexible Energy Harvesters. Adv. Funct. Mater..

[110] Hwang G.-T., Park H., Lee J.-H., Oh S., Park K.-I., Byun M., Park H., Ahn G., Jeong C.K., No K. (2014). Self-Powered Cardiac Pacemaker Enabled by Flexible Single Crystalline PMN-PT Piezoelectric Energy Harvester. Adv. Mater..

[111] Hinchet R., Yoon H.-J., Ryu H., Kim M.-K., Choi E.-K., Kim D.-S., Kim S.-W. (2019). Transcutaneous ultrasound energy harvesting using capacitive triboelectric technology. Science.

[112] Gil B., Li B., Gao A.Z., Yang G.-Z. (2020). Miniaturized Piezo Force Sensor for a Medical Catheter and Implantable Device. ACS Appl. Electron. Mater..

[113] Tang W., Tian J., Zheng Q., Yan L., Wang J., Li Z., Wang Z.L. (2015). Implantable Self-Powered Low-Level Laser Cure System for Mouse Embryonic Osteoblasts’ Proliferation and Differentiation. ACS Nano.

[114] Dong L., Wen C.S., Liu Y., Xu Z., Closson A.B., Han X.M., Escobar G.P., Oglesby M., Feldman M., Chen Z. (2019). Piezoelectric Buckled Beam Array on a Pacemaker Lead for Energy Harvesting. Adv. Mater. Technol..

[115] Li J., Kang L., Long Y., Wei H., Yu Y.H., Wang Y.H., Ferreira C.A., Yao G., Zhang Z.Y., Carlos C. (2018). Implanted Battery-Free Direct-Current Micro-Power Supply from in Vivo Breath Energy Harvesting. ACS Appl. Mater. Interfaces.

[116] Dong L., Closson A.B., Oglesby M., Escobedo D., Han X.M., Nie Y., Huang S.C., Feldman M.D., Chen Z., Zhang J.X.J. (2019). In vivo cardiac power generation enabled by an integrated helical piezoelectric pacemaker lead. Nano Energy.

[117] Dagdeviren C., Yang B.D., Su Y.W., Tran P.L., Joe P., Anderson E., Xia J., Doraiswamy V., Dehdashti B., Feng X. (2014). Conformal piezoelectric energy harvesting and storage from motions of the heart, lung, and diaphragm. Proc. Natl. Acad. Sci. USA.

[118] Curry E.J., Le T.T., Das R., Ke K., Santorella E.M., Paul D., Chorsi M.T., Tran K.T.M., Baroody J., Borges E.R. (2020). Biodegradable nanofiber-based piezoelectric transducer. Proc. Natl. Acad. Sci. USA.

[119] Cheng X.L., Xue X., Ma Y., Han M.D., Zhang W., Xu Z.Y., Zhang H., Zhang H.X. (2016). Implantable and self-powered blood pressure monitoring based on a piezoelectric thinfilm: Simulated, in vitro and in vivo studies. Nano Energy.

[120] Cheng B.L., Ma J.X., Li G.D., Bai S., Xu Q., Cui X., Cheng L., Qin Y., Wang Z.L. (2020). Mechanically Asymmetrical Triboelectric Nanogenerator for Self-Powered Monitoring of In Vivo Microscale Weak Movement. Adv. Energy Mater..

[121] Cheng X.L., Miao L.M., Song Y., Su Z.M., Chen H.T., Chen X.X., Zhang J.X., Zhang H.X. (2017). High efficiency power management and charge boosting strategy for a triboelectric nanogenerator. Nano Energy.

[122] Brenes A., Morel A., Juillard J., Lefeuvre E., Badel A. (2020). Maximum power point of piezoelectric energy harvesters: A review of optimality condition for electrical tuning. Smart Mater. Struct..

